# Emerging roles of mistletoes in malignancy management

**DOI:** 10.1007/s13205-013-0124-6

**Published:** 2013-03-07

**Authors:** Seema Patel, Suryakanta Panda

**Affiliations:** 1 Affiliated to Better Process Control School, Department of Food Science and Technology, University of California, Davis, CA USA; 2 Computer Technology Resources, Irvine, CA USA

**Keywords:** Mistletoe, Anticancer, Lectin, Polysaccharide, Immune modifier

## Abstract

Mistletoes are a group of obligate plant semi-parasites in the order Santalales. These clumps of plants growing on a wide range of host plants have been traditionally regarded as medicinal repositories. However, current scientific discoveries have validated their health potentials like never before. Their extracts containing alkaloids, viscotoxins, lectins, and polysaccharides have been evidenced to possess a myriad biological potentials including cancer inhibition. Mistletoes have emerged as promising alternative therapy against colon, oral, lung, and pancreas cancers. The plant extracts bolster immunity, delay tumour initiation and progression, kill malignant tumours, stabilize DNA, alleviate side effects of chemotherapeutics, improve the lifespan, and coping ability of cancer patients and survivors. A range of proprietary formulations viz. Iscador, Eurixor, Helixor, Lektinol, Isorel, Iscucin, Abnoba-viscum and recombinant lectin ML-1 are already being commercialized. This review presents an informative account on the recent developments in mistletoe-mediated cancer management. The underlying mechanisms, possibilities and limitations in cancer therapeutic development are outlined for kindling both researcher and public interest.

## Introduction

**Fig. 1:**
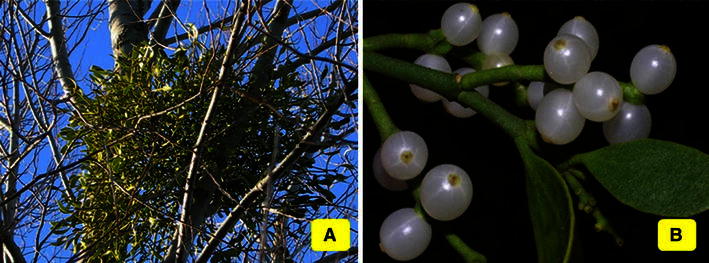
**a** Mistletoe plant on host tree, **b** mistletoe with berries

Mistletoes are semi-parasitic plants belonging to family Misodendraceae, Loranthaceae, Santalaceae, Viscaceae, etc. grouped under the order Santalales. Though, there exist more than 100 species, the most recognized genera are Viscum, Phoradendron, Arceuthobium, Peraxilla, Loranthus, Amylotheca, Amyema, Taxillus, Psittacanthus, Scurrula, etc. The list of mistletoes with documented medicinal roles has been given in Table 1. They are distributed across Europe, America, Asia and Africa to Australia and New Zealand. Depending on their geographical location, the mistletoes are named European, American, Mexican, Korean, African, Japanese, and Indian, etc. It is the state floral emblem of Oklahoma state in the USA. Mistletoes have tiny, oval green leaves on forked branches (Fig. 1). These plants bear flowers that develop into white or red berries. The chlorophylls enable them to carry out photosynthesis, but the nutritional requirement is not satisfied by this meagre carbohydrate produced. So, these plants penetrate their haustoria into the host plants and siphon off the nutrients. They parasitize a wide range of trees, namely, apple, almond, plum, eucalyptus, beech, poplar, spruce, rosewood, maple, sweetgum, oak, mesquite, willow, elm, pine, juniper, etc. These plants have a rich traditional significance. Sprigs of mistletoe are hung as Christmas decorations, as the bouquets are believed to be the harbingers of good luck. In folklore medication, mistletoes have been prolifically used. Several Native American tribes used juniper mistletoe as medication, tea and food. Xhosa people of South Africa used it for amelioration of sore throat and lumbago. The Japanese used it against hypertension and rheumatism. Mistletoes are claimed to exert antioxidant, analgesic, anti-inflammation, immune-stimulatory, antiglycemic, neuroprotective, and antihypertensive properties. Further, the ability to provide relief from diarrhoea, cardiovascular risk, osteoarthritis, osteoporosis, and epilepsy has been documented. Now, results of randomised clinical trials are surfacing, lending credentials to the ethnic uses.

**Table 1:** The common and scientific names of referred mistletoes and the families to which they belong

Mistletoes	Species	Family	References
American Mexican Juniper	*Phoradendron villosum* *Phoradendron leucarpum* *Phoradendron macrophyllum* *Phoradendron serotinum* *Phoradendron brachystachyum* *Phoradendron juniperinum*	Viscaceae	Alonso-Castro et al. (2012) Lopez-Martinez et al. (2013)
European Red-berry Korean	*Viscum album* *Viscum cruciatum* *Viscum coloratum* *Viscum liquidambaricolum*	Loranthaceae	Cebovic et al. (2008) Hong and Lyu (2012) Yang et al. (2011)
Catkin	*Taxillus sutchuenensis* *Taxillus liquidambaricola*	Loranthaceae	Liu et al. (2012)
Mexican	*Psittacanthus calyculatus*	Loranthaceae	Moustapha et al. (2011)
Loranthus	*Scurrula parasitica* L.	Loranthaceae	Xiao et al. (2010)

Recently, their intervention in carcinogenesis has grabbed the interest of the researchers. So far, inhibition against breast, lung, pancreas, gastric cancer, and melanoma has been reported. This review aims to present the major findings in the anti-proliferative properties of mistletoes for integration of these plants in mainstream onco-care practices. The bioactive phytochemicals and the mechanisms involved have been outlined.

## Phytochemical profile

**Fig. 2:**
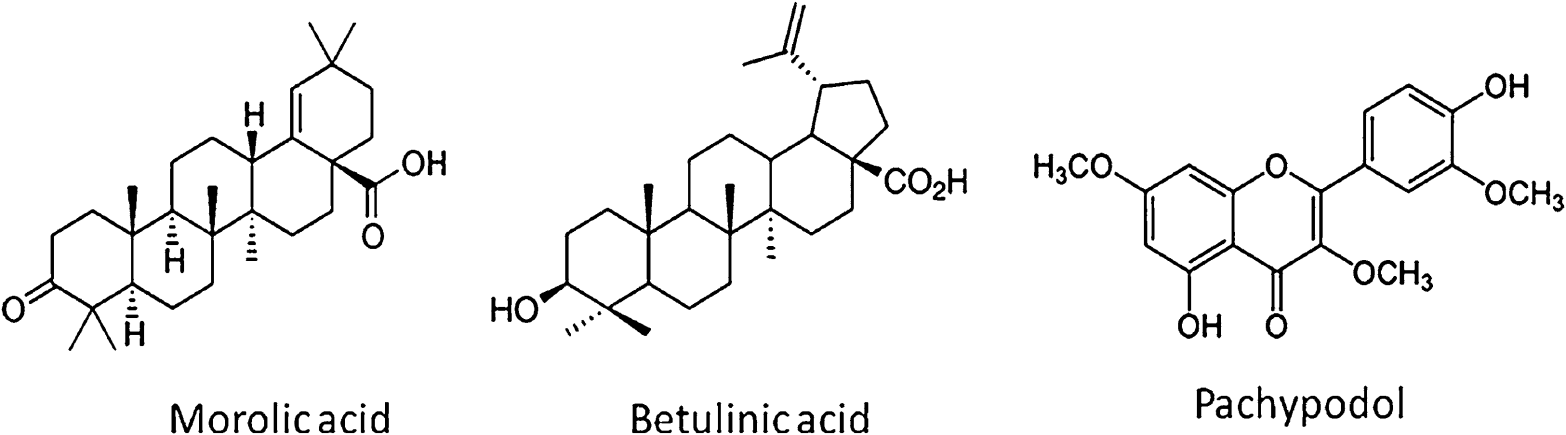
Key anticancer compounds found in mistletoe

A wide repertoire of phytochemicals has been extracted using various solvents and analyzed by chromatographic techniques. Lectins that bind specifically to the carbohydrate moiety of glyco-conjugates are abundant in the mistletoes. Xiao et al. (2010) extracted polysaccharides possible antitumor potential from *Scurrula parasitica*. Herbal tea concocted from mistletoe has the triterpenoids, oleanolic acid and betulinic acid in high concentrations (Jager et al. 2011). Moustapha et al. (2011) identified gallic acid, two flavonol-3-biosides and the non-protein amino acid *N*-methyl-*trans*-4-hydroxy-l-proline from *Psittacanthus calyculatus*, a mistletoe species abundant in Mexico. Yang et al. (2011) isolated and identified triterpenoids and triterpenoid saponins of *V. liquidambaricolum* that exhibited cytotoxic activities against four human tumour cell lines (HeLa, SGC-7901, MCF-7, and U251). The acetone extract of *P. brachystachyum* yielded morolic acid as the major component. Also, β-sitosterol, stigmasterol, triacontanol, squalene, α- and β-amyrin, lupeol, lupenone, a range of aldehydes and phenolic acids were identified from the extracts (Lopez-Martinez et al. 2013). Omeje et al. (2012) isolated lupeol-based triterpenoid esters from the leaves of *L. micranthus* Linn. Zhao et al. (2012) determined the cytotoxic activities of *V. coloratum* flavonoid compounds pachypodol and ombuine against four human tumour cell lines (HeLa, SGC-7901, MCF-7, and U251) and obtained promising results. Liu et al. (2012) evaluated the anti-proliferative activities of the aqueous-ethanol extract of *Taxillus sutchuenensis* on A549 cells. The ethyl-acetate fractions had the highest anti-proliferative activity which is attributed to the phenolic compounds. The major anticancer compounds have been presented in Fig. 2.

## Validated anticancer properties

Many types of mistletoes have demonstrated enhanced cancer surveillance, antitumor, anti-angiogenic, and apoptotic activities. Distinct inhibitory effects have been observed against colon, oral, lung, pancreas cancers. Several commercial preparations are administered as part of complementary onco-therapy. Peritumoral injections are the most common method of mistletoe extract administration. *V. album* is by far the most-studied and promising mistletoe. Several literature reviews have documented the developments from time to time. Laszczyk (2009) reviewed the triterpene-rich mistletoe shoots as possible cancer therapy. Ostermann et al. (2009) reviewed the piling evidences and reported the correlation between Iscador usage and survivability. Melzer et al. (2009) formulated a review on the quality of life improvement by mistletoe preparations. Distillation of the conclusions from control trials and questionnaires revealed the efficacy in suppressing solid tumours. Kienle and Kiene (2010) scoured the databases for deriving the impact of *V. album* on the quality of life of cancer patients. Both randomized and non-randomized control trials were assessed. Studies with methodological precision showed better coping, fatigue, sleep, exhaustion, energy, nausea, vomiting, appetite, depression, anxiety, ability to work and emotional and functional well-being. Further, it was well tolerated and found to alleviate the side effects of chemotherapy and radiation.

Zwierzina et al. (2011) have reviewed the anti-proliferative role of recombinant lectin aviscumine. Fu et al. (2011) attributed the anticancer property of mistletoe lectins to the modulation of the programmed cell death pathways. Olaku and White (2011) reviewed the clinical trials in cancer populations. Metelmann et al. (2012) reviewed the impact of mistletoe extract in direct contact with the tumour tissue and held activation of macrophage polarization followed by induced cytotoxicity as the inhibitory pathway.

## Commercial anticancer preparations

The National Center for Complementary and Alternative Medicine under the aegis of National Cancer Institute constantly searches for new, effective anticancer drugs. Till now an array of mistletoe extracts have been formulated as drugs, namely Iscador, Eurixor, Helixor, Lektinol, Isorel, Iscucin, Abnoba-viscum and recombinant lectin ML-1. The extracts are applied as injection into skin, vein, pleural cavity or tumour. Friedel et al. (2009) evaluated the efficacy of treatment with mistletoe extract Iscador in non-metastatic colorectal carcinoma patients through a cohort study. These results suggest the beneficial effect of Iscador therapy. Iscador was well tolerated without life-threatening adverse reactions, drug interactions, or tumour enhancement. Gren (2009) also studied the influence of Iscador Qu, M and P (5 mg/kg) on the total protein concentration in blood serum and proportions of blood protein fractions. A significant increase in albumin fraction level and lymphocyte count was observed that was assumed to be reason of enhanced immunity.

## Research findings

This review strives to furnish the key developments in the current times. Monira et al. (2009) examined the effect of lectin from *V. album* subsp. *coloratum* on cytokine gene expression in human colon adenocarcinoma Caco-2 cells and in the mouse intestine. The results indicated the up-regulation of the gene expression of the proinflammatory cytokines interleukin (IL)-8, tumor necrosis factor-alpha (TNF-α) and IL-6 in Caco-2 cells and TNF-α and IL-6 in the duodenum. (Kirsch and Hajto 2011) studied the effect of lectins on sarcoma patients. When the optimal dose of 0.75–1.0 ng/kg lectin was given twice a week subcutaneously, remissions of tumor symptoms were conspicuous. Lectins functioning as ligands for pattern recognition receptors of the natural immune systems are docked to ganglioside molecules (CD75) of monocytes and granulocytes, stimulating the natural antitumor mechanisms. Kameda et al. (2011) observed the effect of 30 months of mistletoe therapy on a patient diagnosed with nodal large cell ALK-1-anaplastic lymphoma compounded with lymphomatoid papulosis. After the designated period, complete remission of the tumor was seen. Park et al. (2012) investigated the effect of Abnobaviscum F^®^ formulated from *V. album* extract on the growth and survival of different leukaemia cell lines. The treatment reduced survival and induced apoptosis of human myeloid leukaemia K562, human plasmacytoma RPMI-8226 and murine lymphocytic leukaemia L1210 cells in culture. The apoptosis mechanism was intrinsic, associated with the activation of caspase-9, JNK-1/2 and p38 MAPK, as well as with the down-regulation of Mcl-1, and inhibition of ERK-1/2 and PKB phosphorylation.

## Antimutagenic

Safeguarding against mutagens is of major importance in cancer prevention at which mistletoe extracts have been fairly effective. Burkhart et al. (2010) carried out an investigation on the protective effect of mistletoe extract on human peripheral blood mononuclear cells of healthy blood donors and the T cell leukaemia Jurkat cell line against the alkylating agent 4-hydroperoxycyclophosphamide. The cells being incubated with the extract for 60–65 h were exposed to the alkylating agent for 2 h. The extract exerted protection towards only healthy cells and not towards the Jurkat cells. The biological action was evident from the enhanced mitochondrial activity and replication of the healthy cells. Hong and Lyu (2012) investigated the antimutagenic potential of *V. album* L. var. *coloratum* agglutinin. The *Salmonella typhimurium* strain TA98 was subjected to the mutagens 2-aminoanthracene and furylfuramide; whereas *Salmonella typhimurium* strain TA100 was subjected to sodium azide and 2-aminoanthracene. The protective ability varied from moderate to negligible. Sekeroglu and Sekeroglu (2012) investigated the cyto-genotoxicity lowering effects of pre-treatment with *V. album* extract on methotrexate-induced chromosomal aberrations in mouse bone-marrow cells. Pre-treatment of mice by gavaging with the extract at the dose 250 mg/kg/day for 10 days caused a significant decrease in chromosomal aberrations and in the number of aberrant cells with the mutations.

## Anti-angiogenesis, antiproliferation, and apoptosis

Angiogenesis, proliferation, and apoptosis are interlinked processes during carcinogenesis. Once mutation occurred and tumour was initiated, delaying its spread is the next challenge to tackle. Ma et al. (2008) investigated the effect of Chinese mistletoe lectin (CML-55) on colon cancer cell line CT26-bearing BALB/c mice. Results showed that compared to PBS treated mice, CML-55 treated group showed significantly delayed colon cancer progression. Enhancement in the tumour antigen-specific activation and proliferation of CD4+ and CD8+ T cells was observed. Also, increase in the NK cell numbers was seen. Promotion of both innate and adaptive immunity projects the extract as an anticancer candidate. Role of mistletoe at this phase have been investigated with appreciable results. The EA hy926 cell line is a continuous human cell line that displays a number of features characteristic of vascular endothelial cell. When plated on a matrigel, they undergo a process of morphological re-organization mimicking angiogenesis. Elluru et al. (2009) determined the cytotoxicity of *V. album* extract on matrigel-treated EA-hy926 cells both in vitro and in vivo. The extract caused significant inhibition of angiogenesis by bringing many changes in vessel formation. Choi et al. (2012) analyzed the proliferation suppressive effect *V. album* subsp. *coloratum* lectin on HepG2Acells. A dose as low as 1–5 pg/ml dose could exert cancer cell-specific toxicity. Alonso-Castro et al. (2012) evaluated cytotoxicity of ethanolic extracts of *P. serotinum* injected intraperitoneally into C57BL/6 mice implanted with TC-1 cells for 25 consecutive days. The extract at the dose 10 mg/kg inhibited the tumour growth by 69 %, increasing the release of IL-2, IL-6, and IFN-γ. Podlech et al. (2012) showed that Iscador Q reduced the migratory and invasive potential of glioblastoma cells. It delayed tumor growth by NK-cell-mediated glioblastoma cell lysis. Cyclodextrin solubilized triterpene acid- or lectin-containing extracts of mistletoe inhibited cell proliferation and demonstrated cytotoxic properties on acute lymphoblastic leukaemia NALM-6 cells in vitro. Furthermore, caspase activity demonstrated that these extracts were able to induce apoptosis in the cancer cells through both caspase-8 and -9 dependent pathways. The treatment of mice with the extract prolonged mean survival to 50.5 days compared to 39.3 days in the phosphate-buffered saline group (Delebinski et al. 2012).

## Post-surgery supportive care

Mistletoe extracts have been found to confer amelioration to post-surgery cancer patients. Fatigue decrease and overall quality of life improvement are the benefits imparted. Elsasser-Beile et al. (2005) observed the effect of intravesical administration of mistletoe lectin to post-surgery superficial urothelial bladder carcinomas patients. After transurethral resection, each patient received mistletoe extracts with lectin concentrations between 10–5,000 ng/ml. The injected extract was retained in the bladder for 2 h. Within the observation time of 12 months, 70 % patients remained tumour-free. The lectin-rich extract might be a suitable alternative to the prevalent BCG therapy, without the side-effects associated with the latter. Wode et al. (2009) investigated the impact of mistletoe extracts on cancer-related fatigue. A patient with recurrent breast cancer history of 10 years was given this plant extract. A two and half year continuation of the drug exerted a dose-dependent benefit in decreasing fatigue. Matthes et al. (2010) conducted a multicenter, controlled observational study to determine the adjuvant chemotherapy with gemcitabine supported by Iscador in the pancreatic carcinoma patients after surgery. With symptom control, overall rise in survival was observed. Eisenbraun et al. (2011) evaluated the effect of aqueous mistletoe extracts on health-related quality of life of breast cancer patients on chemotherapeutics. Questionnaires pertaining the therapeutic value of the extract abnobaVISCUM(^®^) Mali were administered to the patients. Self-assessment was analyzed at four different phases of the regimen. Tolerability was recorded very well for 91 % of the patients and the efficacy was rated as good or very good for 94 %. 89 % of the patients reported about a good or very good benefit. Brandenberger et al. (2012) administered a questionnaire to cancer patients suffering from different types of cancers at the beginning and after 3 months of mistletoe administration. Analysis of the interview revealed higher vitality and autonomy, better ability to cope with the cancer in the patients undergoing mistletoe therapy. Büssing et al. (2012) employed a random-effect meta-analysis to find out the effect of Iscador and observed short-time beneficial psychosomatic effect. Kim et al. (2012) evaluated safety and efficacy of a standardized mistletoe extract abnobaVISCUM(^®^) in operated patients with gastric cancer. The effect of oral therapy with doxifluridine combined with subcutaneous injection of mistletoe extract three times per week from postoperative day 7 to week 24 in increasing doses was monitored. Health status, leukocyte and eosinophil counts increased significantly and diarrhoea incidences were low.

## Drug side-effect amelioration

Troger et al. (2009) carried out a prospective randomized study to determine the immunostimulant property of Iscador^®^ M in breast cancer patients. Conventional cancer drugs viz. cyclophosphamide, adriamycin and 5-fluoro-uracil led to neutropenia (abnormal decrease in the number of neutrophils). The chemotherapy-induced neutropenia amelioration effect of the mistletoe-based drug was studied. The treated group showed an improvement in the neutrophil counts. Bar-Sela et al. (2012) administered chemotherapy plus Iscador to patients with non-small-cell lung cancer. Though survival time was similar in both the groups, the chemotherapy dose reductions, severe non-haematological side-effects and hospitalizations were less frequent in patients treated with Iscador.

## Synergistic effects

Several instances of mistletoe potentiating chemical drugs exist. Freudlsperger et al. (2010) investigated the synergistic antiproliferative efficacy of mistletoe lectin-I and the PPAR-γ ligand rosiglitazone in malignant melanoma cells. Results of the XTT cell proliferation assay showed that the combined therapy is more effective than individual treatment. Metastatic pancreatic is a fatal disease with brief median survival time of 3–6 months only. Oxaliplatin and gemcitabine are the standard chemotherapy to combat the cancer. Ritter et al. (2010) investigated the adjunct therapy possibilities with mistletoe extract. In a case study, 37 weeks after surgery, the patient demonstrated a sustained partial remission, and the chemotherapy was stopped. Contrary to normal trend, the patient showed no signs of tumour progression, even 10 months later. The long-term remission was attributed to the mistletoe therapy initiated after the surgery. Micke et al. (2010) conducted a questionnaire-based investigation on the usage of CAM in lung cancer patients. 54 % of the patients reported using CAM out of which 15 % used mistletoe. Iscador, the aqueous preparation of *V. album* L. is regarded a potent CAM. Sabova et al. (2010) investigated the cytotoxic and apoptosis-inducing effects of aqueous mistletoe extract alone or in combination with doxorubicin on Jurkat cells. Dose-dependent DNA fragmentation was induced in Jurkat cells individually as well as synergistically.

## Mechanisms of action

**Fig. 3:**
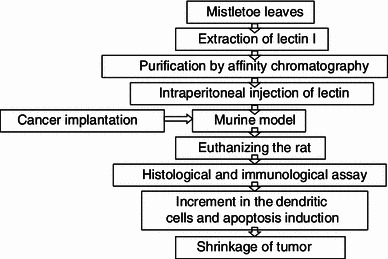
Schematic diagram of mistletoe lectin-mediated anticancer activity

Cebovic et al. (2008) employed supercritical CO_2_ extraction coupled with gas chromatography/mass spectrometry for selective extraction and identification of therapeutic compounds from *V. album* leaves. The extract exhibited in vivo cytotoxicity towards Ehrlich carcinoma (EAC) cells due to the induction of oxidative stress. Kovacs et al. (2008) compared the effect of *V. album* extract and the alkaloid vincristine on B cell lymphoma cell line WSU-1 in an in vitro system. Both the substances first inhibited the proliferation of the tumour cells which lead to apoptosis or necrosis. Reduction in the DNA synthesis of the G2/M cell cycle phase was decoded to the molecular pathway followed. Both the substances led to dose-dependent apoptosis at 12 h as well as 24 h. Xiao et al. (2010) investigated the antitumor activity of *Scurrula parasitica* leaf polysaccharides. Intraperitoneal injection of the polysaccharide inhibited S180 growth with a tumor inhibition rate of 54 %. Analyses by immunohistochemistry techniques showed that the polysaccharide down-regulates the expression of Ki-67, CyclinD1, and Bcl-2 protein, and up-regulates the expression of Bax protein. Li et al. (2011) investigated the effect of CM-1, a lectin-I extracted from the Chinese mistletoe on colorectal cancer cells. The in vitro and in vivo effect on CLY and HT-29 cells were studied. Using miRNA microarray assay and qRT-PCR analysis, the anticancer action was decoded due to precursor degradation-mediated down-regulation of some miRNAs by CM-1. The miR-135a and miR-135b were the miRNAs most down-regulated. Strüh et al. (2012) solubilised the oleanolic acid-rich triterpene extract from mistletoe by 2-hydroxypropyl-β-cyclodextrin on B16.F10 mouse melanoma cells. The solubilised extract showed high cytotoxicity by causing DNA fragmentation, followed by loss of membrane integrity and intracellular adenosine-5′-triphosphate. Ucar et al. (2012) demonstrated that pretreatment with the methanolic extract of *V. album* before heat shock at 40 °C increased apoptosis via caspase-3 activation (60 %) in C6 glioma cells. This down-regulation of the expression of Hsp27 and 14-3-3 chaperone proteins was revealed to induce apoptosis. A schematic illustration of the anticancer activity of lectin has been presented in Fig. 3.

## Side effects and future trends

FDA is yet to approve the mainstream use of mistletoe in cancer therapy on the grounds of undesirable side-effects. Some mistletoe extracts caused several types of human cancer cells to grow faster. The major adverse effects documented so far are inflammation, headaches, fever, chills, and anaphylactic shocks.

Chemical makeup profiling is required. The nexus between phytochemicals in the semi-parasite and the host tree needs further verification. Efficacy of different species, harvest time and fermentation period are the areas to be addressed. Feeble water solubility of triterpenes hinders the water extraction. Cyclodextrins have improved their solubilizing property resulting in better biological activity. Safety issues often blight the prospect of pharmaceutical use of mistletoe extracts. Accidental ingestion has been reported to cause gastric upset, seizure, eye irritation, etc. Complaints of inflammation at the site of subcutaneous injection have been recorded that must be verified. Huber et al. (2011) conducted a 3-armed randomized, double-blind clinical trial on healthy volunteers by giving them subcutaneous injections of various doses of the drug Iscucin^®^ Populi (IP) twice a week over a period of 12 weeks. Immunological assays showed eosinophilia and an increase of CD4 cells but not an increase of the tumor causing cytokine IL-6 negating doubts of safety. Still, animal studies, and human trials are required to reach any concrete conclusions. Heterogeneity of test animals often gives rise to variation in results. So, collection of epidemiological data is desired. The synergistic effect with other anticancer agents needs to be explored. The dosage needs to be standardized. The results pooled till now are not consistent due to small sample size and methodological heterogeneity. More accurate designs need to be planned for reliable results.

## Conclusions

The validated reports support the previous ethno-pharmacological relevance of mistletoes. The effective outcomes ask for further assessment of the antiproliferative potential of the extracts. Many species are threatened that must be preserved. Side effects cause conflict in broader prescription as adjunct therapy that must be addressed. Safety parameters and proper dosage need to be ascertained. Existing evidences may not be enough but given due research impetus, a novel drug with comparable or superior efficacy than paclitaxel, vincristine, epipodophyllotoxin, and betulinic acid may emerge. Promotion of mistletoe as complementary and alternative medicine (CAM) will certainly usher in a new hope for cancer therapy. It is certainly the time to view this poorly recognized botanical resource in new light for which this review is expected to be a catalyst.
